# Supplementary material for: Metabolic perceptrons for neural computing in biological systems
Source: Nat Commun. 2019 Aug 28;10:3880. doi: 10.1038/s41467-019-11889-0 (PMC6713752; doi:10.1038/s41467-019-11889-0)
Supplement: Supplementary file 1 — Supplementary Information [file 41467_2019_11889_MOESM1_ESM.pdf]

Supplementary information for:

Metabolic Perceptrons for Neural Computing in Biological Systems

Pandi and Koch *et al.*

The supplementary information contains supplementary figures and tables:

Supplementary Figure 1. Feedback-loop circuit design of the benzoate actuator.
Supplementary Figure 2. Comparison of the maximum signal of whole-cell circuits.
Supplementary Figure 3. 2D plots for the data presented in the heatmap in Figure 2b.
Supplementary Figure 4. Examining the effect of resource competition versus enzyme efficiency on the whole-cell cocaine transducer.
Supplementary Figure 5. Examining the effect of resource competition versus enzyme efficiency on the whole-cell metabolic adder.
Supplementary Figure 6. The specific growth rate (μ) values of the whole-cell circuits presented in Figure 1.
Supplementary Figure 7. The specific growth rate (μ) values of the whole-cell adder presented in Figure 2.
Supplementary Figure 8. The dose-response of cell-free transducers to different concentrations of the associated enzymes DNA (weights) for weighted transducers.
Supplementary Figure 9. Weighted transducers model results for experimental results presented in Figure 4.
Supplementary Figure 10. Five different binary classification problems using a metabolic perceptron for hippurate and cocaine.
Supplementary Figure 11. Model simulations for classifiers in Figure 6.
Supplementary Figure 12. Further characterization of HipO enzyme (hippurate transforming enzyme) at lower concentrations of the enzyme.
Supplementary Figure 13. Exploring Hippurate-Cocaine on-off behavior with different weights and input concentrations for hippurate.
Supplementary Figure 14. Strategies for multi-layer perceptron implementation.
Supplementary Figure 15. Simulations from the random sampling of estimated parameters in whole-cell system.
Supplementary Figure 16. Simulations from the random sampling of estimated parameters in the cell free system.

Supplementary Table 1. Goodness of fit scores for the whole-cell models.
Supplementary Table 2. Goodness of fit scores for the cell-free models.
Supplementary Table 3. Parameter estimations for whole-cell model.
Supplementary Table 4. Parameter estimations for cell-free model.
Supplementary Table 5. List of sequences and their source used in this study.
Supplementary Table 6. List of plasmids used in this study deposited to Addgene.
Supplementary Table 7. The mean and standard deviation of the normalized data of whole-cell and cell-free data plotted in all figures and supplementary figures, and model simulated/predicted results associated with each experiment, also submitted as separate Source Data excel file.

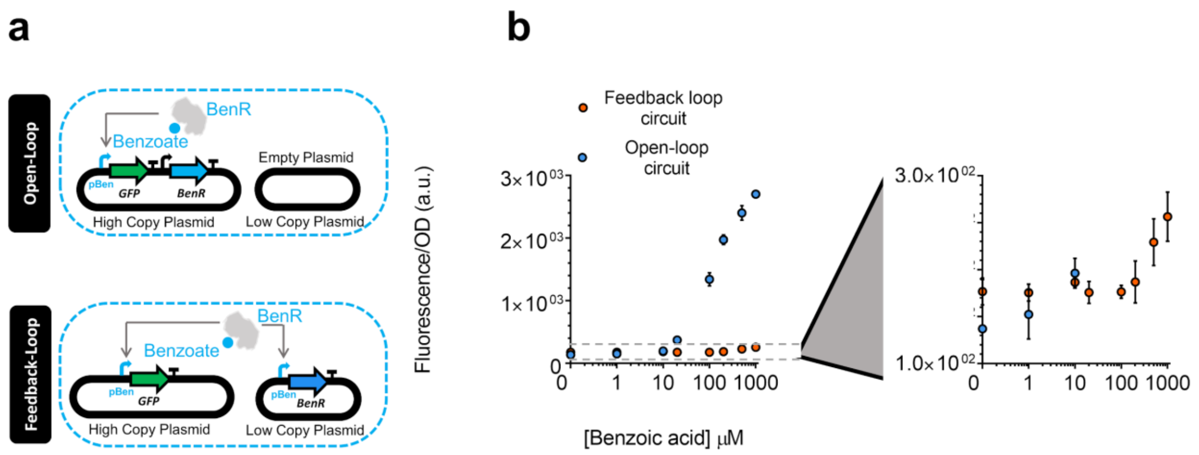

Supplementary Figure 1. Feedback-loop circuit design of the benzoate actuator. (**a**) The open-loop circuit (**Figure 1b**) versus a feedback-loop circuit for the benzoate actuator. In the feedback-loop actuator the gene encoding TF is expressed under its responsive promoter, pBen, in a low copy plasmid and sfGFP reporting the signal in a high copy plasmid[1]. (**b**) The dose-response of the feedback-loop versus the open-loop circuit (**Figure 1c**) to different concentrations of benzoate. All data points and the error bars are the mean and standard deviation of normalized values from measurements taken from three different colonies on the same day.

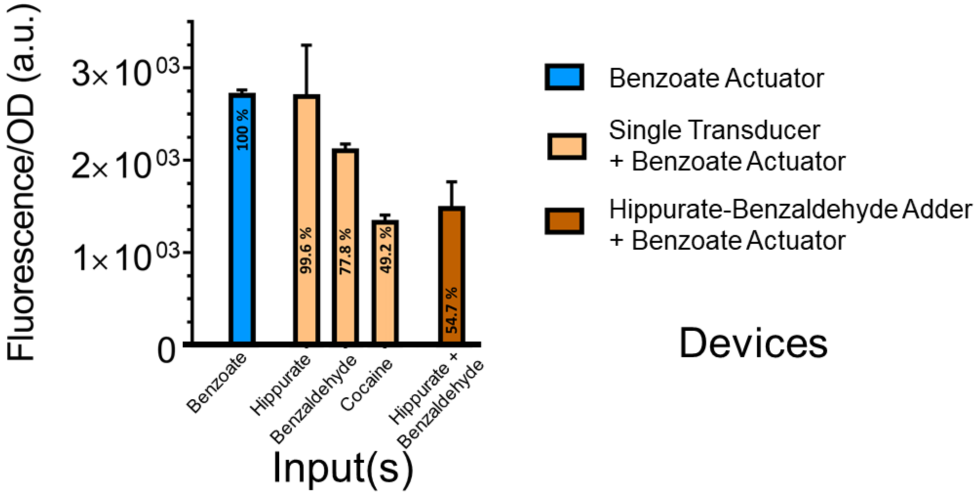

Supplementary Figure 2. Comparison of the maximum signals of whole-cell circuits. Comparison of the maximal signal of hippurate, benzaldehyde, and cocaine transducers (beige) as well as hippurate-benzaldehyde adder (orange) with benzoate actuator (blue). The maximum signal of all the circuits are at the maximum concentration of their inputs (1000 µM). The percentage in each bar represents its value with regard to the maximum signal of benzoate in benzoate actuator. The actuator (blue) and transducer (beige) data and error bars are from the results presented in **Figure 1**. The adder (orange) data and error bars are from the results presented in **Figure 2**.

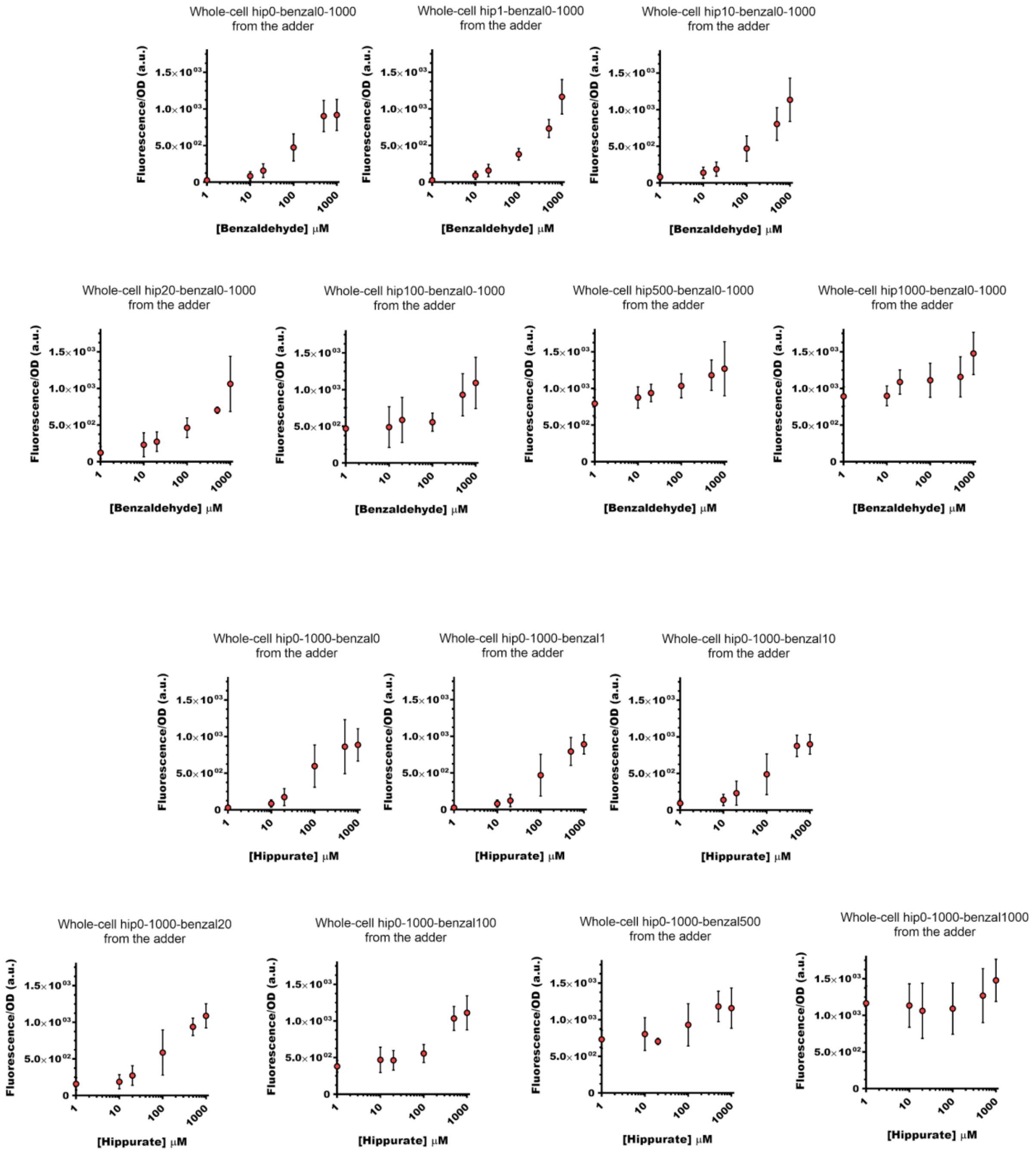

Supplementary Figure 3. 2D plots for the data presented in heatmap in Figure 2b. These 14 plots help visualize the linearity of metabolic addition. At the top of each plot the columns/rows corresponding to the heatmap in **Figure 2b** have been labelled.

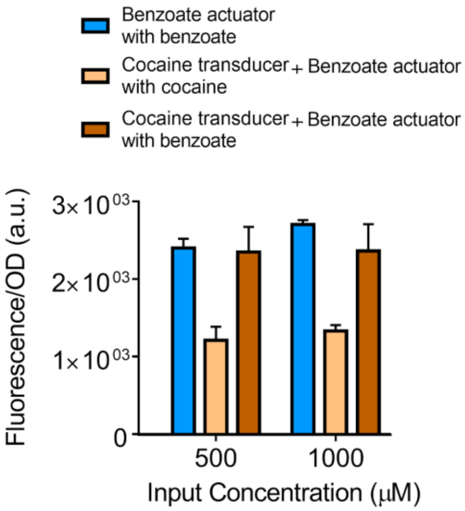

Supplementary Figure 4. Examining the effect of resource competition versus enzyme efficiency on the whole-cell cocaine transducer. To study these effects on the single-enzyme metabolic circuit, the following experiment was performed: cocaine transducer (with the highest signal dissipation among the three tested in **Figure 1**) was supplied with benzoate input, to test the effect of enzymes on only cellular resource allocation but not the conversion of inputs to benzoate. The cocaine transducer (+ benzoate actuator) with benzoate input shows a behavior similar or close to the benzoate actuator alone. All data points and the error bars are the mean and standard deviation of normalized values from measurements taken from three different colonies on the same day.

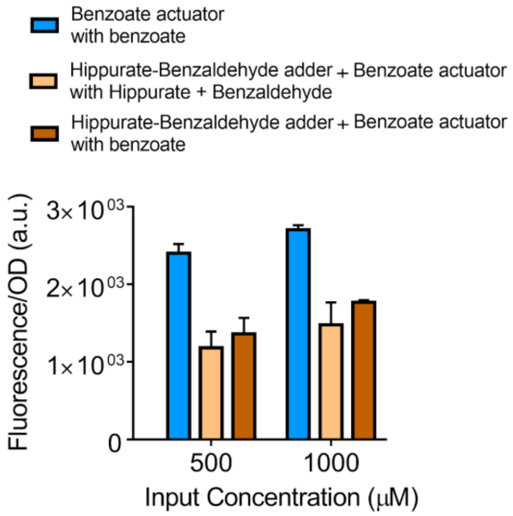

Supplementary Figure 5. Examining the effect of resource competition versus enzyme efficiency on the whole-cell metabolic adder. To study these effects on the two-enzyme metabolic circuit (adder) the following experiment was performed: hippurate-benzaldehyde adder was supplied with benzoate input, to test the effect of enzymes on only cellular resource allocation but not the conversion of inputs to benzoate. The adder (+ benzoate actuator) with benzoate input shows a behavior similar to the adder (+ benzoate actuator) with hippurate and benzaldehyde inputs. All data points and the error bars are the mean and standard deviation of normalized values from measurements taken from three different colonies on the same day.

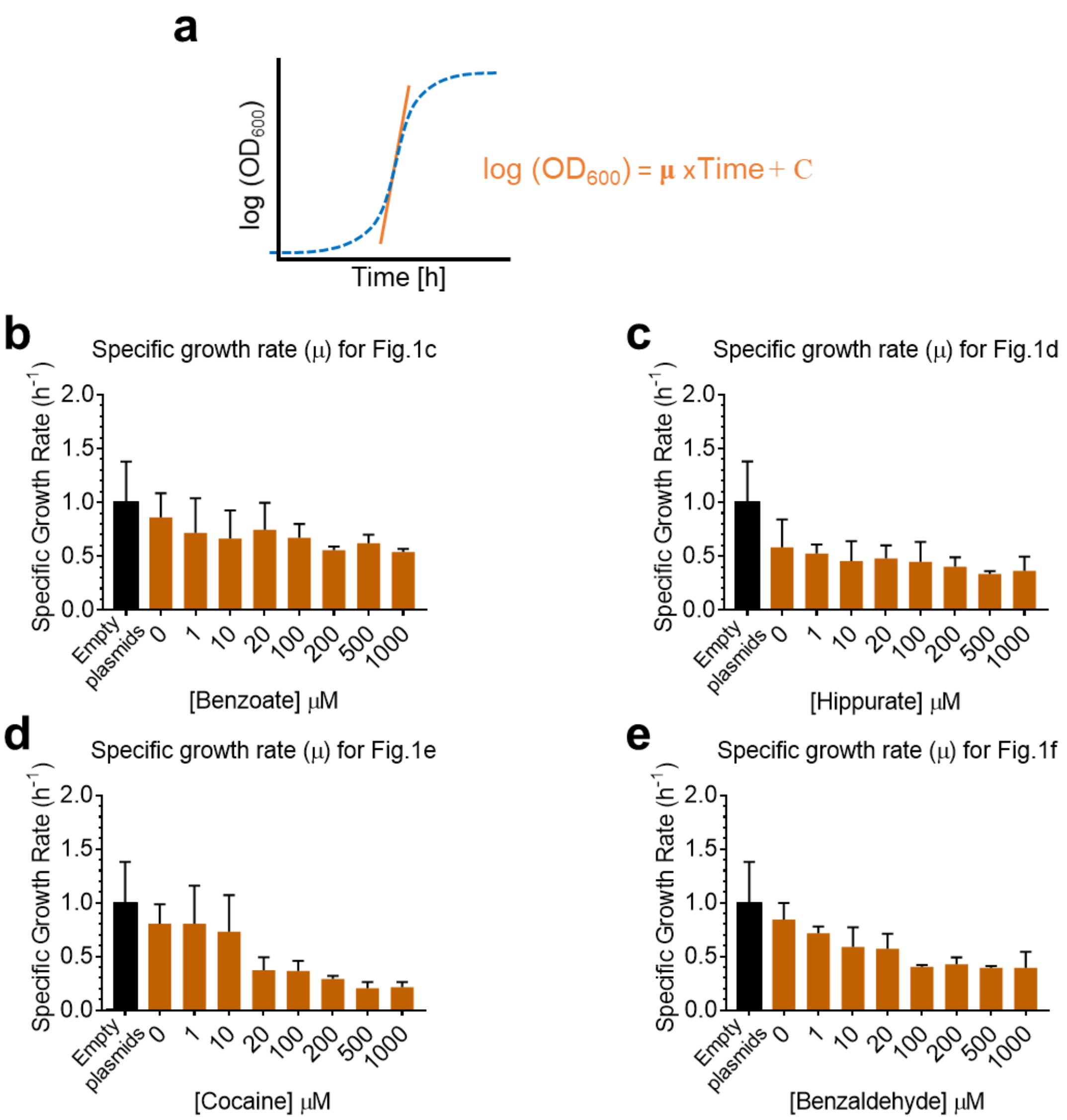

Supplementary Figure 6. The specific growth rate (μ) values of the whole-cell circuits presented in Figure 1. (**a**) The schematic of the calculation of the specific growth rate (μ) values from OD_{600} kinetic values over time. It is calculated as the slope of the line drawn in the range of exponential phase of the growth when log (OD_{600}) is plotted over time. The specific growth rate (μ) values of the cells harboring circuits for benzoate actuator (**b**), hippurate (**c**), cocaine (**d**) and benzaldehyde (**e**) transducers presented in **Figure 1**. The OD data were collected from cells exposed to the input metabolite for 2-4 hours and growing at 37 °C in a 96-well plate using a plate reader (Biotek Synergy HTX). All data points and the error bars are the mean and standard deviation of normalized values from measurements taken from three different colonies on the same day.

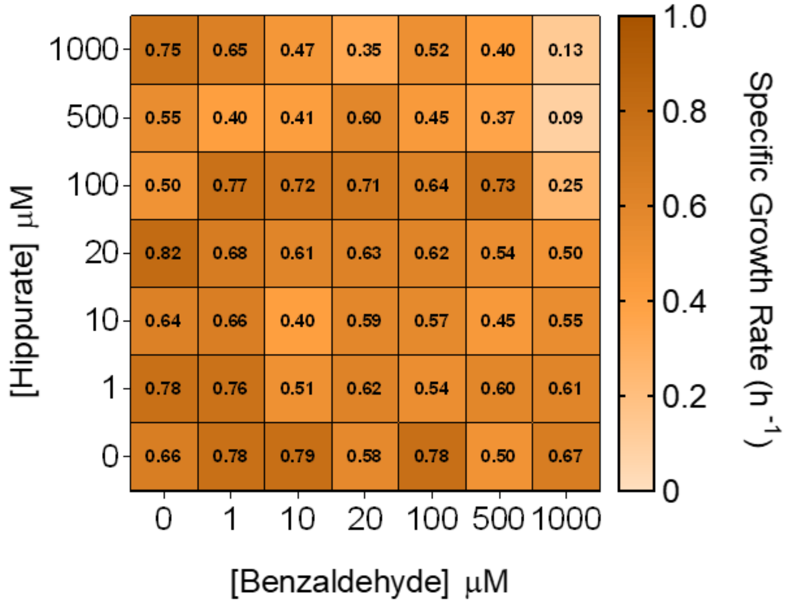

Supplementary Figure 7. The specific growth rate (μ) values of the whole-cell adder presented in Figure 2b. The specific growth rate (μ) values for the adder presented in **Figure 2b**. The OD data were collected from cells exposed to the input metabolites for 2-4 hours and growing at 37 °C in a 96-well plate using a plate reader (Biotek Synergy HTX). The schematic of the calculation of the specific growth rate (μ) values from OD_{600} kinetic values over time is presented in **Supplementary Figure 6a**. It is calculated as the slope of the line drawn in the range of the exponential phase of growth when log (OD_{600}) is plotted over time. All data points are the mean of normalized values from measurements taken from three different colonies on the same day.

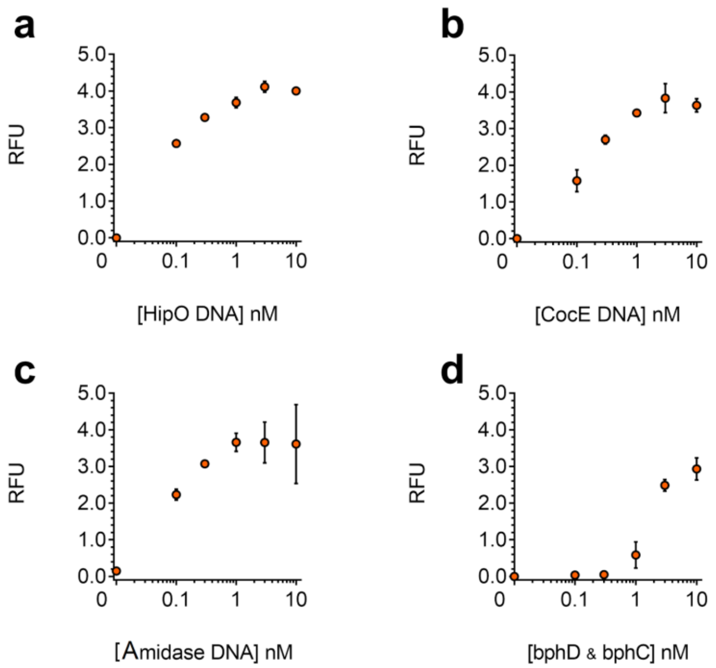

Supplementary Figure 8. The dose-response of cell-free transducers to different concentrations of the associated enzyme DNAs (weights) for weighted transducers. The behavior of the cell-free transducers at constant concentration of inputs (100 µM) while the weights (concentration of the enzyme DNAs) are varied for hippurate (**a**), cocaine (**b**), benzamide (**c**) and biphenyl-2,3-diol (**d**) transducers. These are plotted using the data in the third column of the heatmaps in **Figure 4** as the average, and the error bars as SD from measurements taken from three independent cell-free reactions on the same day (RFU: Relative Fluorescence Unit).

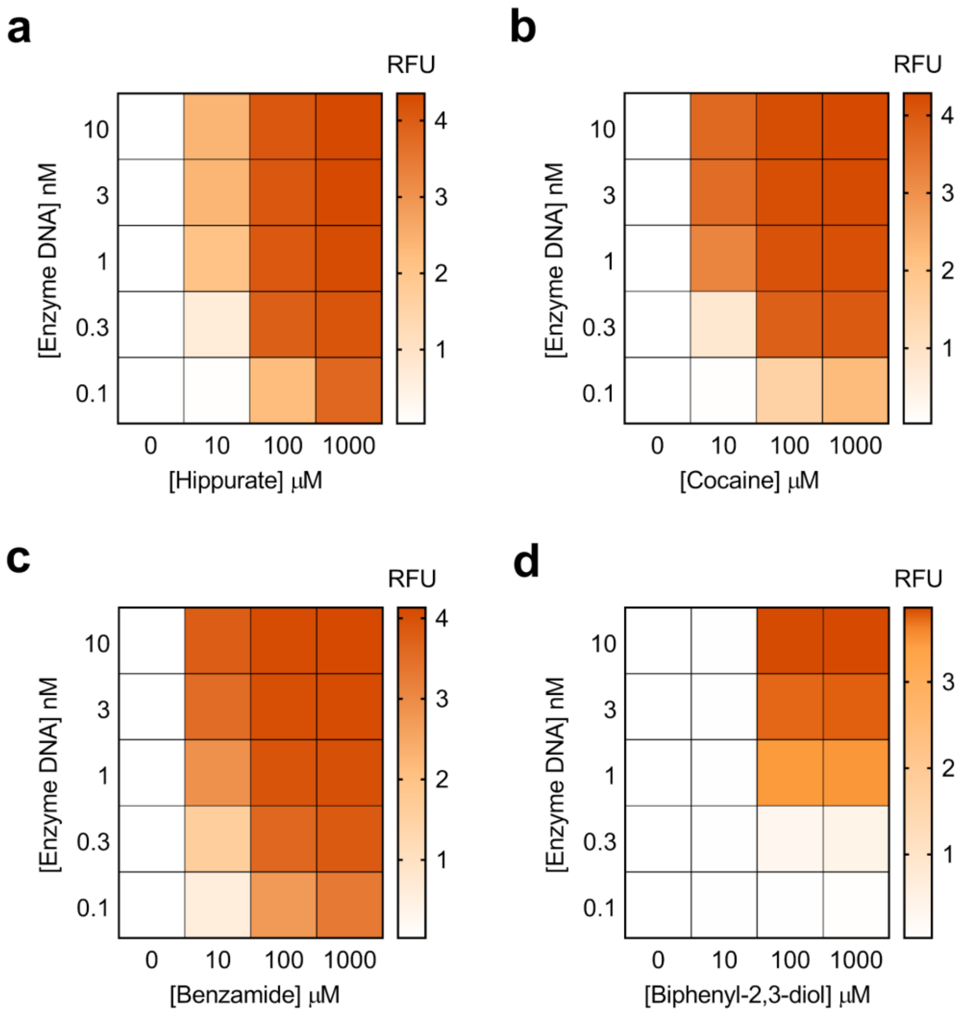

Supplementary Figure 9. Weighted transducers model results. The model simulations for experimental conditions presented in **Figure 4**. (**a,b,c,d**) Heatmaps representing model simulations for weighted transducers at different concentrations of input molecules and enzymes DNA for hippurate (**a**), cocaine (**b**), benzamide (**c**) and biphenyl-2,3-diol (**d**). (RFU: Relative Fluorescence Unit)

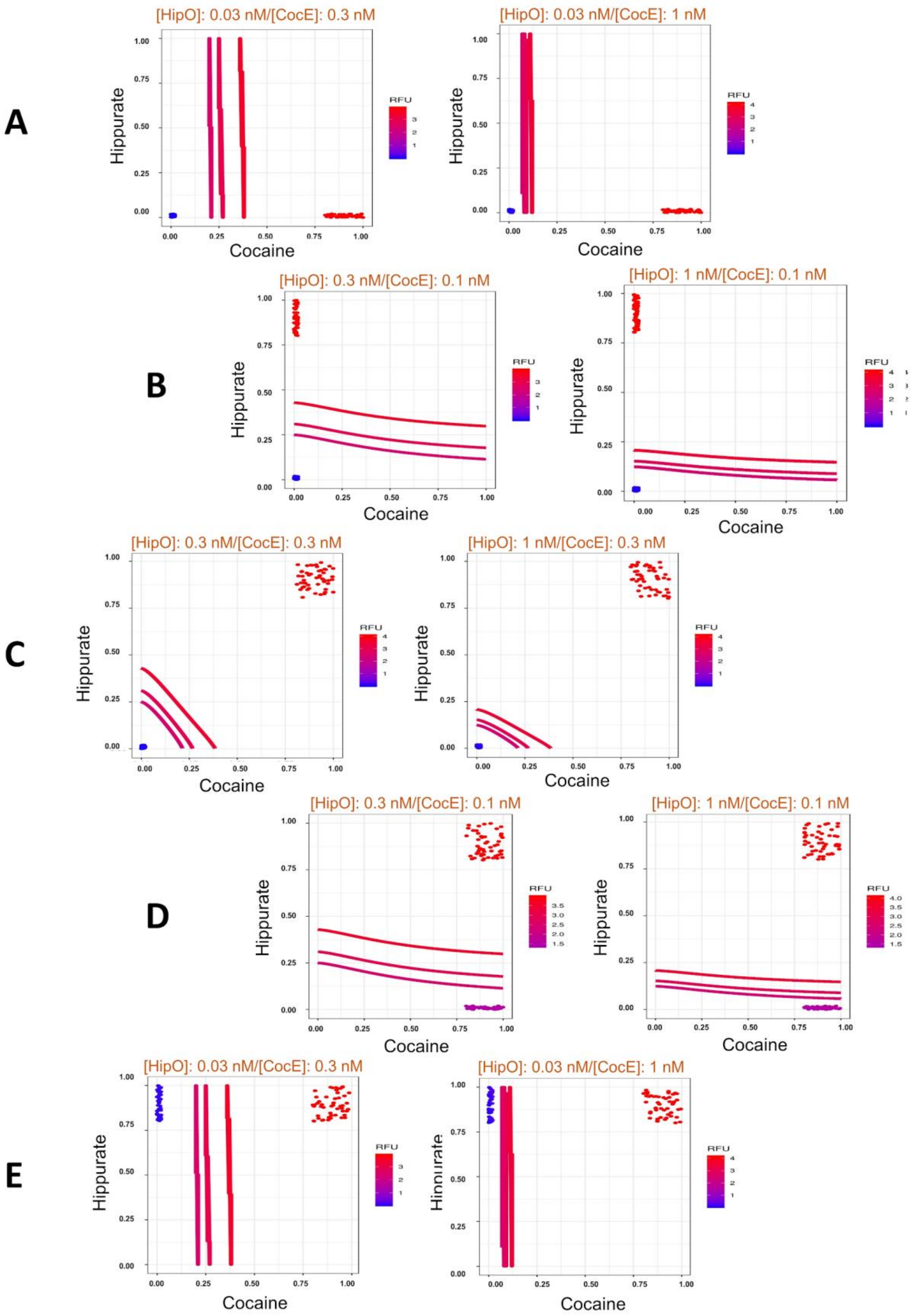
A
[HipO]: 0.03 nM/[CocE]: 0.3 nM
[HipO]: 0.03 nM/[CocE]: 1 nM
B
[HipO]: 0.3 nM/[CocE]: 0.1 nM
[HipO]: 1 nM/[CocE]: 0.1 nM
C
[HipO]: 0.3 nM/[CocE]: 0.3 nM
[HipO]: 1 nM/[CocE]: 0.3 nM
D
[HipO]: 0.3 nM/[CocE]: 0.1 nM
[HipO]: 1 nM/[CocE]: 0.1 nM
E
[HipO]: 0.03 nM/[CocE]: 0.3 nM
[HipO]: 0.03 nM/[CocE]: 1 nM
Hippurate
Cocaine
RFU
0.00
0.25
0.50
0.75
1.00

Supplementary Figure 10. Five different binary classification problems using a metabolic perceptron for hippurate and cocaine. (**A** to **E**). For each problem, the scatter plot shows multiple data points that represent a combination of input values of cocaine and hippurate. The concentrations for those points are sampled between 0 and 2 µM for low values and 80 and 100 µM for high values. The data points in each problem belong to two different sets that can be separated by a threshold line into two separate clusters. The trained model is then used to identify weights needed to be applied to the weighted transducers such that a decision threshold 'd' classifies the two clusters into red (ON, >d) or blue (OFF, <= d). The threshold lines shown in the plots represent three iso-fluorescence lines that successfully classify the data into the binary categories: ON and OFF. (RFU: Relative Fluorescence Unit)

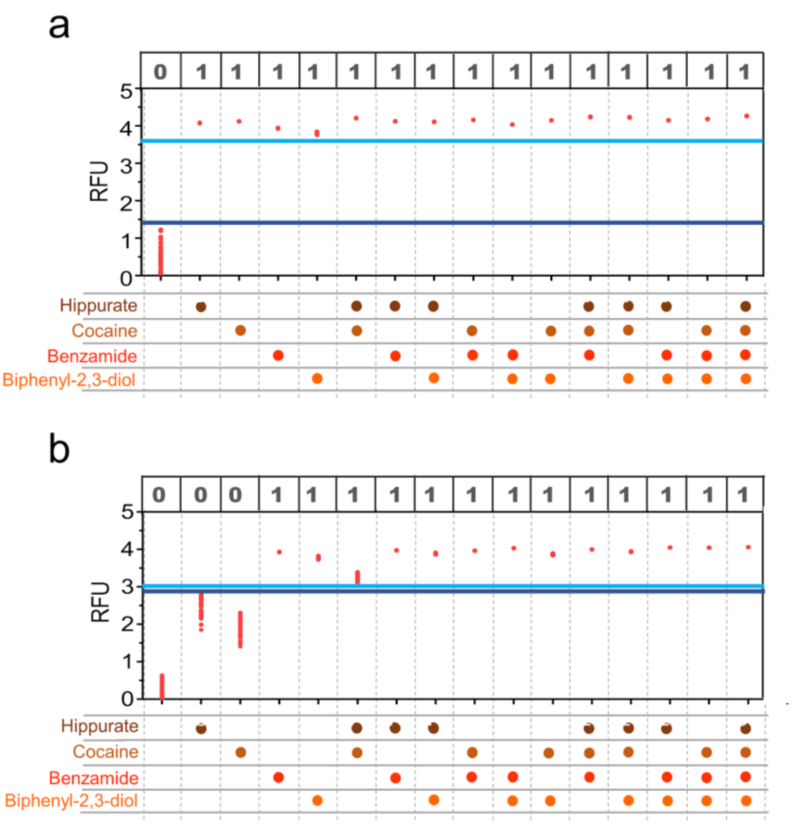

Supplementary Figure 11. Model simulations for classifiers in Figure 6. Predictions associated with (**a**) the full OR classifier (**Figure 6c**) and (**b**) the first calculation for "[cocaine *(C)* AND hippurate *(H)*] OR benzamide *(B)* OR biphenyl-2,3-diol *(F)*" classifier with 0.1 nM HipO weight with (instead of 0.03 as experimentally tested and presented in **Figure 6d**). In order to perform the clusterings, we sampled values uniformly within the stated ranges ([0, 2µM] for low values and [80, 100µM] for high values). We then simulated the results to assess the robustness of our designs. Two blue lines refer to the thresholds separating "OFF" and "ON" states. The panel of "OFF" and "ON" at the top of the plots are the expected outputs. (RFU: Relative Fluorescence Unit).

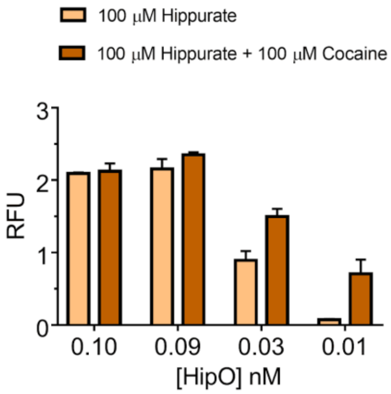

Supplementary Figure 12. Further characterization of HipO enzyme (hippurate transforming enzyme) at lower concentrations of the enzyme and 100 µM hippurate. HipO enzyme which for its weight led to higher signals than predicted, needed to be further characterized at concentrations lower than the minimum concentration used for the weighted metabolic circuits (0.1 nM). For this characterization, this figure shows the effect of 100 µM hippurate input alone and its additive effect when coupled with 100 µM cocaine at the weight (CocE enzyme concentration) of 0.1 nM. All data are the mean and the error bars are the standard deviation of normalized values from measurements taken from two or three independent cell-free reactions on the same day. (RFU: Relative Fluorescence Unit).

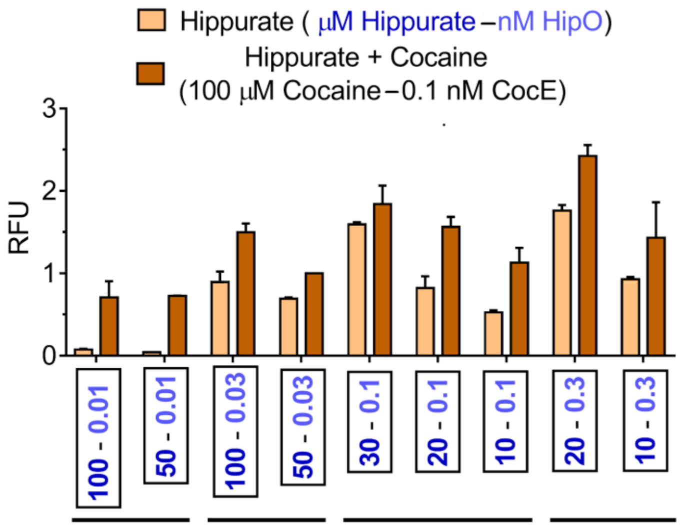

Supplementary Figure 13. Exploring Hippurate-Cocaine ON-OFF behavior with different weights and input concentrations for hippurate. All these experiments were done while Cocaine is at a concentration of 100 µM and weight of 0.1 nM CocE. The beige bars are for hippurate (µM Hippurate – nM HipO) and the orange bars are for Hippurate (µM Hippurate – nM HipO) + Cocaine (100 µM Cocaine – 0.1 nM CocE) as inputs. All data are the mean and the error bars are the standard deviation of normalized values from measurements taken from two independent cell-free reactions on the same day. (RFU: Relative Fluorescence Unit).

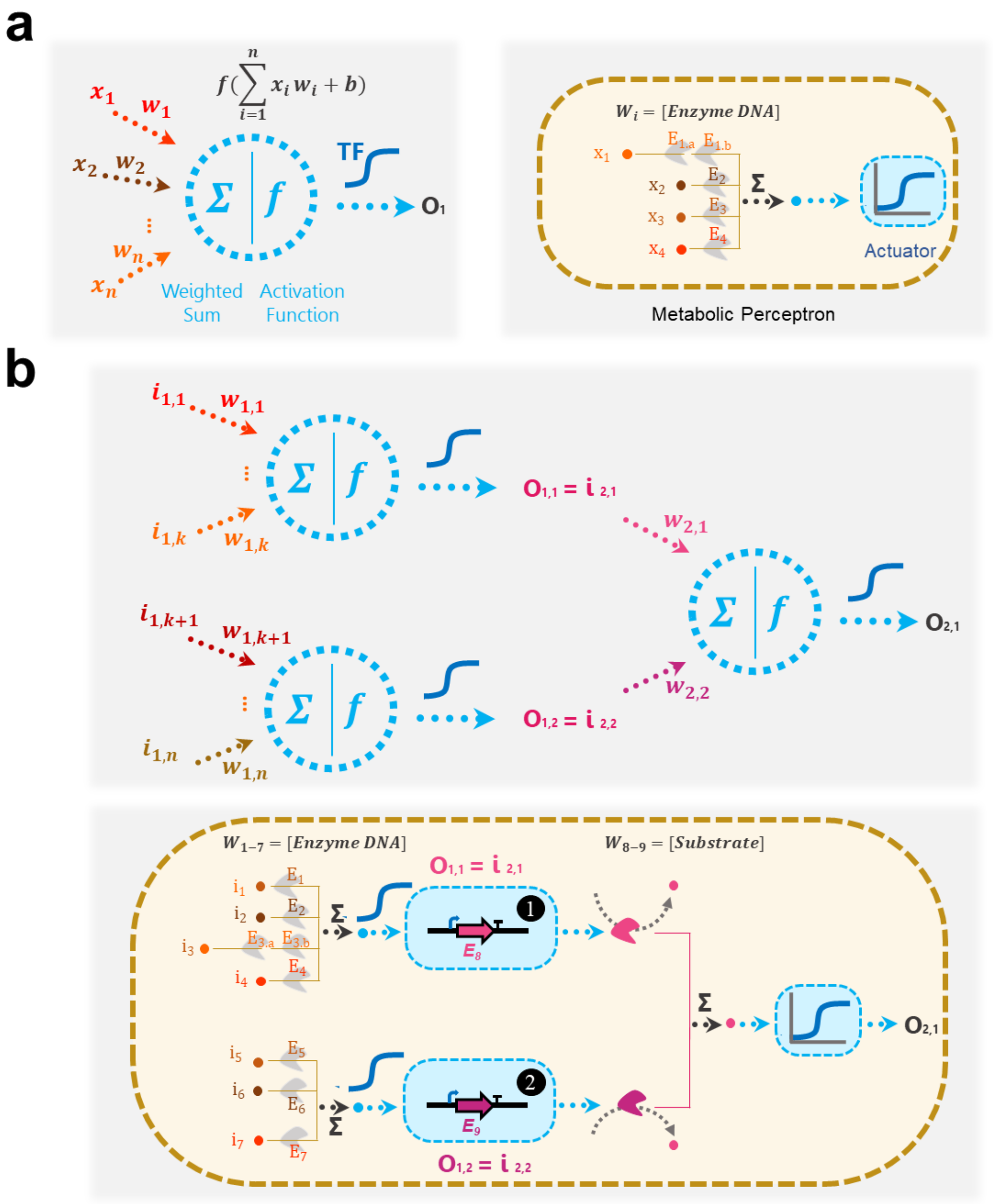

Supplementary Figure 14. Strategies for multi-layer perceptron implementation. (a) *Left:* The schematic presents how computation is performed in a single-layer perceptron: inputs (x_{i-n}) are converted into a common metabolite using enzymes that allow for weighting (w_i) each input (x_i) individually. The common metabolite is then converted into output O_1 using a non-linear activation layer (using a transcription factor =TF). *Right:* A single-layer metabolic perceptron composed of multiple input metabolites (x_{1-4}) and metabolic enzymes (E_{1-4}) transforming the inputs into a common metabolite. The common metabolite then activates the gene expression, representing the actuator function. **(b)** The schematic presents how computation is performed in a multi-layer perceptron (Top) and a possible implementation of a multi-layer metabolic

perceptron (Bottom). In a multi-layer perceptron, the outputs of the first perceptron layer are used as inputs for the second layer. We suggest a potential strategy for such implementation. (1) A TF actuator outputs enzyme E8 ($O_{1,1}$) from the first layer that behaves as an input ($I_{2,1}$) for the second layer, in turn producing a metabolite needed as effector in the next perceptron layer. (2) Similarly, another TF actuator outputs enzyme E9 ($O_{1,2}$) from the first layer that behaves as an input ($I_{2,2}$) for the second layer, also producing the same effector metabolite needed in the next perceptron layer. Weights on the second perceptron layer can be applied by tuning the concentrations of the substrate metabolites for E8 and E9. This strategy is the converse of what we did in the first layer, where enzyme DNA concentrations were weights and input metabolites were ‘0’ or ‘1’. Here, the enzymes E8 and E9 are ‘0’ or ‘1’, as they are outputs from sigmoidal functions, whereas the metabolite concentrations are the weights. I

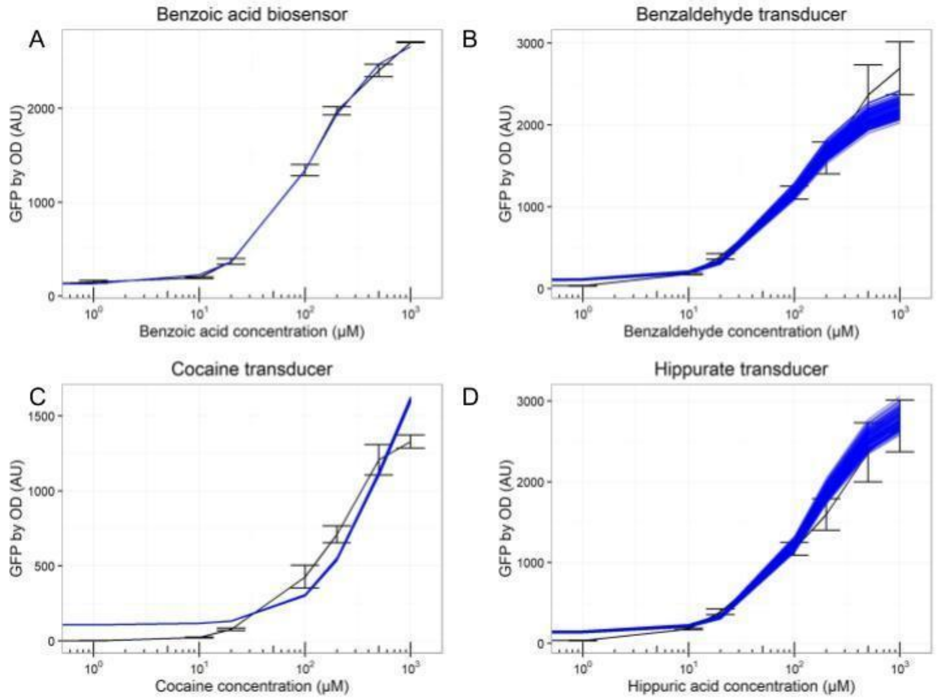

Supplementary Figure 15. Simulations from the random sampling of estimated parameters in whole-cell system. Representation of the experimental data with SEM (n = 3) in black, and in blue, the results from 100 simulations of the model with parameters drawn from the final parameters estimation without refitting. The combination of various parameters within our estimations correctly recapitulates the data. (A) benzoate actuator, (B) benzaldehyde transducer, (C) cocaine transducer, and (D) hippurate transducer. Scripts provided in GitHub also allow for visualization of those results for each axis of the adder in Figure 2.

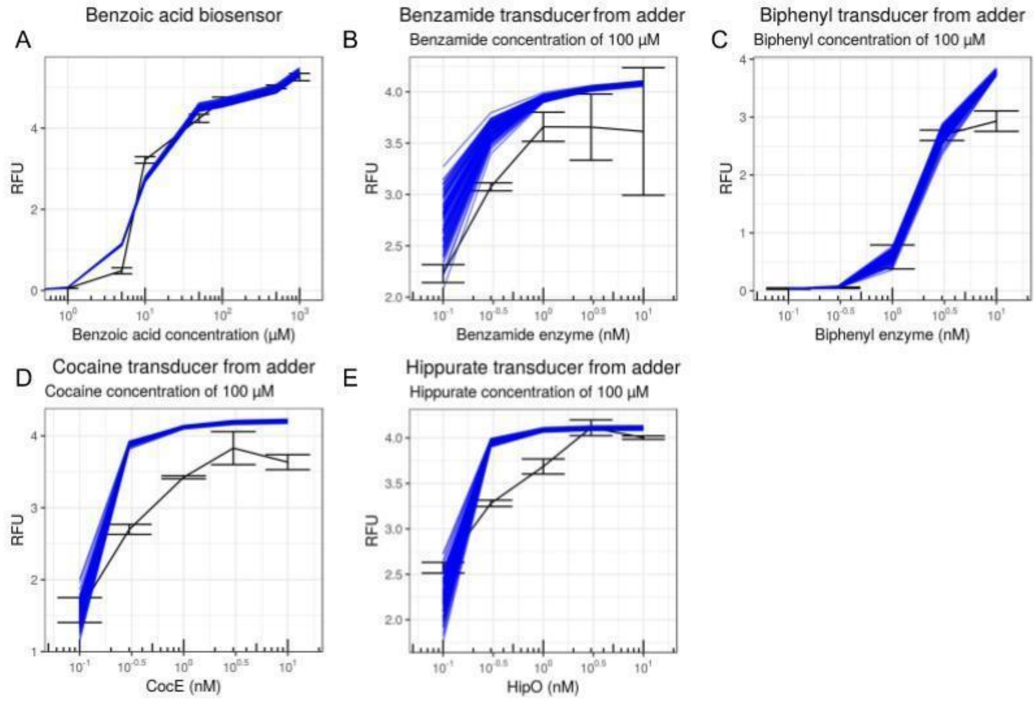

Supplementary Figure 16. Simulations from the random sampling of estimated parameters in the cell-free system. Representation of the experimental data with SEM (n = 3) in black, and in blue, the results from 100 simulations of the model with parameters drawn from the final parameters estimation without refitting. The combination of various parameters within our estimations correctly recapitulates the data. (A) benzoate actuator, (B) benzamide transducer, (C) biphenyl-2,3-diol transducer, (D) cocaine transducer, and (E) hippurate transducer. The simulation of the transducers were performed with 100 µM of the input metabolites as will be used in the classifier experiments. Scripts provided in GitHub also allow for the visualisation of those results for other axis of the various heatmaps in Figure 4. (RFU: Relative Fluorescence Unit).

Supplementary Table 1. Goodness of fit scores for the whole-cell models.

Score	Correlation	Weighted R squared	R squared	Error percentage	Fit or prediction
**Actuator**	0.999	0.999	0.999	NA	Fit
**Benzaldehyde transducer**	0.995	0.992	0.980	NA	Fit
**Hippurate Transducer**	0.997	0.990	0.983	NA	Fit
**Cocaine Transducer**	0.965	0.950	0.924	NA	Fit
**Adder - complete**	0.958	0.982	0.916	16.8 %	Fit (on inducer = 0) and prediction
**Adder - both inputs present**	0.947	0.931	0.889	15.3 %	Prediction

The correlation (from the R cor function), Weighted R squared and R squared between the experimental data and the model. Exact definition of the weighted R squared and the R squared are provided in the Methods section, as well as the RMSD that is used to compare models.

Supplementary Table 2. Goodness of fit scores for the cell-free models.

Score	Correlation	Weighted R squared	R squared	Error percentage	Fit or prediction
**Actuator**	0.990	0.999	0.980	NA	Fit
**Cocaine Transducer**	0.923	0.999	0.574	NA	Fit
**Hippurate Transducer**	0.984	0.999	0.962	NA	Fit
**Benzamide Transducer**	0.946	0.991	0.659	NA	Fit
**2,3 biphenyl Transducer**	0.965	0.998	0.762	NA	Fit
**Fixed enzyme Adder**	0.910	0.998	0.653	10.1%	Prediction
**Fixed inducer adder**	0.919	0.986	0.784	16.0%	Prediction
**Full OR classifier**	0.973	0.980	0.823	9%	Prediction
**(C AND H) OR B Or F- Fig6**	0.985	0.999	0.913	16.9 %	Prediction

Supplementary Table 3. Parameter estimations for in vivo model.

**Parameter**	**Mean Value +- 95 Confidence Interval**
Hill_a	1.34 +- 1 e-6
Km	114 +- 1 e-4
Fc	20.6 +- 3 e-5
Basal	130 +- 2 e-4
Range_BenZ	1.1 +- 1 e-6
Range_HipO	0.787 +- 1 e-6
Range_CocE	0.201 +- 2.97 e-3
E	4.22 +- 0.193
Ratio	0.776 +- 3.7 e-3
nr	1.956 +- 4.56 e-2
Range_res	1.973 +- 0.107

Mean value plus and minus 95% Confidence Interval.

Supplementary Table 4. Parameter estimations for cell-free model.

Parameter	Mean Value +- 95 CI
Hill_a	2.2 +- 0.1
Km	8.40 +- 9 e-3
Fc	137 +- 1.84 (sd : 9.41)
Basal	3.29 e-2 +- 4 e-4 (sd : 2 e-3)
Lin	8.19 +- 9.3 e-2
Range_HipO	488 +- 35
K_HipO	0.396 +- 0.022
K_hippurate	245 +- 29
n_HipO	1.82 +- 0.052
n_hippurate	1.205 +- 0.046
Range_CocE	337 +- 28
K_CocE	0.799 +- 0.00017
K_cocaine	54 .4 +- 5.04
n_CocE	1.713 +- 0.055
n_cocaine	1.44 +- 0.047

range_benzamid_enz	234 +- 20
K_benzamid_enz	3.73 +- 0.27
K_benzamid	48.6 +- 5.5
n_benzamid_enz	0.683 +- 0.072
n_benzamid	0.906 +- 0.087
range_biphenyl_enz	63.7 +6- 4.79
K_biphenyl_enz	8.63 +- 0.31
K_biphenyl	56.3 +- 4.92
n_biphenyl_enz	1.25 +- 0.067
n_biphenyl	3.05 +- 0.192

Mean value plus and minus 95% Confidence Interval (Standard Deviation for fold change and baseline).

Supplementary Table 5. List of sequences and their source used in this study.

Sequence	Description//Nucleotide sequence
**BenR**<br><br>*UniProtKB - Q9L7Y6*<br><br>Taken from Libis et al.[2]	Transcription factor for benzoate, an activator from *Pseudomonas putida*[3]<br><br>ATGGAATCTCGTCTGCTGTCTGAACGTTCTTCTGTTTTCCACCACGCTGACCCGTACGCTGTTTCTGACTACGTTAA<br>CCAGCACGTTGGTCAGCACTGCATCGGTCTGTCTCGTACCACCCACCCGCAGGCTTCTCTGTCTCACCGTAAATTC<br>GCTGAACTGGACCTGTGCCGTATCTCTTACGGTGGTTCTGTTCGTGTTACCTCTCCGGCTCTGGAAACCATCTACC<br>ACCTGCAGGTTCTGCTGAACGGTAACTGCCTGTGGCGTGGTCACAAACGTGAACAGCACCTGGTTCCGGGTGAAC<br>TGCTGCTGATCAACCCGGACGACCCGGTTGACCTGACCTACTCTGAAGACTGCGAAAAATTCATCCTGAAAGTTCC<br>GACCCGTCTGCTGGACTCTATCTGCGACGAACAGCGTTGGCAGCGTCCGGACGGTGGTGTTCGTTTCCTGCGTAA<br>CCACTACCGTCTGGACGAACTGGACGGTTTCGTTAACCTGCTGGCTATGGTTTGCCACGAAGCTGAAGTTTCTGAC<br>TCTCTGCCGCGTGTTCAGGGTCACTACTCTCAGATCGTTGCTTCTAAACTGCTGACCCTGATGTCTACCAACATCCG<br>TCGTGAATCTCTGTCTGCTCCGCAGGCTGGTCTGGAACGTATCCTGGACTACATCGAACGTAACCTGAAACTGGAA<br>CTGTCTGCTGAAGTTCTGGCTGAACAGGCTTGCATGTCTCTGCGTTCTCTGTACGCTCTGTTCGACCAGCACCTGG<br>GTATCACCCCGAAACACTACGTTCGTCAGCGTAAACTGGAACGTGTTCACGCTTGCCTGTCTGACCCGACCTGCGG<br>TGTTCGTTCTGTTACCGAACTGGCTCTGGACTACGGTTTCCTGCACCTGGGTCGTTTCTCTGAAATCTACCGTCAGC<br>AGTTCGGTGAACTGCCGTCTCAGACCTTCAAACGTCGTGCTTAA
**pBen**<br><br>Taken from Libis et al.[2]	Promoter responsive to benzoate-BenR<br><br>ACTGTTCGAAGCATTGCCATTTTCTGAAGTTACCGAAAAAGTACCGAACATCCGTAAATCTGGATAACGTTCTGCAC<br>AATCCGGATAGCCCCCCGCCAGCCGTCTCCCTAACCTGACCAGGTCTAAACAATAACAAGGGAGAGTCTGGCCAT<br>G
**Superfolder GFP (sfGFP)**	ATGCGTAAAGGCGAAGAGCTGTTCACTGGTGTCGTCCCTATTCTGGTGGAACTGGATGGTGATGTCAACGGTCATA<br>AGTTTTCCGTGCGTGGCGAGGGTGAAGGTGACGCAACTAATGGTAAACTGACGCTGAAGTTCATCTGTACTACTGG<br>TAAACTGCCGGTACCTTGGCCGACTCTGGTAACGACGCTGACTTATGGTGTTCAGTGCTTTGCTCGTTATCCGGAC<br>CATATGAAGCAGCATGACTTCTTCAAGTCCGCCATGCCGGAAGGCTATGTGCAGGAACGCACGATTTCCTTTAAGG<br>ATGACGGCACGTACAAAACGCGTGCGGAAGTGAAATTTGAAGGCGATACCCTGGTAAACCGCATTGAGCTGAAAG<br>GCATTGACTTTAAAGAAGACGGCAATATCCTGGGCCATAAGCTGGAATACAATTTTAACAGCCACAATGTTTACATC<br>ACCGCCGATAAACAAAAAAATGGCATTAAAGCGAATTTTAAAATTCGCCACAACGTGGAGGATGGCAGCGTGCAGC<br>TGGCTGATCACTACCAGCAAAACACTCCAATCGGTGATGGTCCTGTTCTGCTGCCAGACAATCACTATCTGAGCAC<br>GCAAAGCGTTCTGTCTAAAGATCCGAACGAGAAACGCGATCATATGGTTCTGCTGGAGTTCGTAACCGCAGCGGG<br>CATCACGCATGGTATGGATGAACTGTACAAATGATGA
**HipO**<br><br>*UniProtKB - P45493*<br><br>Taken from Libis et al.[2]	Hippurate hydrolase (**EC**: 3.5.1.32), *Campylobacter jejuni*<br>Hippurate to benzoate<br><br>ATGAACCTGATCCCGGAAATCCTGGACCTGCAGGGTGAATTCGAAAAAATCCGTCACCAGATCCACGAAAACCCGG<br>AACTGGGTTTCGACGAACTGTGCACCGCTAAACTGGTTGCTCAGAAACTGAAAGAATTCGGTTACGAAGTTTACGA<br>AGAAATCGGTAAAACCGGTGTTGTTGGTGTTCTGAAAAAAGGTAACTCTGACAAAAAAATCGGTCTGCGTGCTGACA<br>TGGACGCTCTGCCGCTGCAGGAATGCACCAACCTGCCGTACAAATCTAAAAAAGAAAACGTTATGCACGCTTGCGG<br>TCACGACGGTCACACCACCTCTCTGCTGCTGGCTGCTAAATACCTGGCTTCTCAGAACTTCAACGGTGCTCTGAAC<br>CTGTACTTCCAGCCGGCTGAAGAAGGTCTGGGTGGTGCTAAAGCTATGATCGAAGACGGTCTGTTCGAAAAATTCG<br>ACTCTGACTACGTTTTCGGTTGGCACAACATGCCGTTCGGTTCTGACAAAAAATTCTACCTGAAAAAAGGTGCTATG<br>ATGGCTTCTTCTGACTCTTACTCTATCGAAGTTATCGGTCGTGGTGGTCACGGTTCTGCTCCGGAAAAAGCTAAAGA<br>CCCGATCTACGCTGCTTCTCTGCTGATCGTTGCTCTGCAGTCTATCGTTTCTCGTAACGTTGACCCGCAGAACTCTG<br>CTGTTGTTTCTATCGGTGCTTTCAACGCTGGTCACGCTTTCAACATCATCCCGGACATCGCTACCATCAAAATGTCT<br>GTTCGTGCTCTGGACAACGAAACCCGTAAACTGACCGAAGAAAAAATCTACAAAATCTGCAAAGGTATCGCTCAGG<br>CTAACGACATCGAAATCAAAATCAACAAAAACGTTGTTGCTCCGGTTACCATGAACAACGACGAAGCTGTTGACTTC<br>GCTTCTGAAGTTGCTAAAGAACTGTTCGGTGAAAAAAACTGCGAATTCAACCACCGTCCGCTGATGGCTTCTGAAG<br>ACTTCGGTTTCTTCTGCGAAATGAAAAAATGCGCTTACGCTTTCCTGGAAAACGAAAACGACATCTACCTGCACAAC<br>TCTTCTTACGTTTTCAACGACAAACTGCTGGCTCGTGCTGCTTCTTACTACGCTAAACTGGCTCTGAAATACCTGAA<br>ATAA

**CocE**<br><br>*UniProtKB - Q9L9D7*<br><br>Taken from Libis et al.[2] and BsaI site removed	Cocaine esterase (**EC**: 3.1.1.84), *Rhodococcus sp.*<br>Cocaine to benzoate<br><br>ATGGTTGACGGTAACTACTCTGTTGCTTCTAACGTTATGGTTCCGATGCGTGACGGTGTTCGTCTGGCTGTTGACCT<br>GTACCGTCCGGACGCTGACGGTCCGGTTCCGGTTCTGCTGGTTCGTAACCCGTACGACAAATTCGACGTTTTCGCT<br>TGGTCTACCCAGTCTACCAACTGGCTGGAATTTGTTCGTGACGGTTACGCTGTTGTTATCCAGGACACCCGTGGTC<br>TGTTCGCTTCTGAAGGTGAATTTGTTCCGCACGTTGACGACGAAGCTGACGCTGAAGACACCCTGTCTTGGATTTT<br>GGAACAGGCTTGGTGCGACGGTAACGTTGGTATGTTCGGTGTTTCTTACCTGGGTGTTACCCAGTGGCAGGCTGC<br>TGTTTCTGGTGTTGGTGGTCTGAAAGCTATCGCTCCGTCTATGGCTTCTGCTGACCTGTACCGTGCTCCGTGGTAC<br>GGTCCGGGTGGTGCTCTGTCTGTTGAAGCGCTGCTGGGTTGGTCTGCTCTGATCGGTACCGGTCTGATCACCTCT<br>CGTTCTGACGCTCGTCCGGAAGACGCTGCTGACTTCGTTCAGCTGGCTGCTATCCTGAACGACGTTGCTGGTGCT<br>GCTTCTGTTACCCCGCTGGCTGAACAGCCGCTGCTGGGTCGTCTGATCCCGTGGGTTATCGACCAGGTTGTTGAC<br>CACCCGGACAACGACGAATCTTGGCAGTCTATCTCTCTGTTCGAACGTCTGGGTGGTCTGGCTACCCCGGCTCTGA<br>TCACCGCTGGTTGGTACGACGGTTTCGTTGGTGAAAGCCTGCGTACCTTCGTTGCTGTTAAAGACAACGCTGACGC<br>TCGTCTGGTTGTTGGTCCGTGGTCCCACTCTAACCTGACCGGTCGTAACGCTGACCGTAAATTCGGTATCGCTGCT<br>ACCTACCCGATCCAGGAAGCTACCACCATGCACAAAGCTTTCTTCGACCGTCACCTGCGTGGTGAAACCGACGCAC<br>TTGCTGGTGTTCCGAAAGTTCGTCTGTTCGTTATGGGTATCGACGAATGGCGTGACGAAACCGACTGGCCGCTGCC<br>GGACACCGCTTACACCCCGTTCTACCTGGGTGGTTCTGGTGCTGCTAACACCTCTACCGGTGGTGGTACCCTGTCT<br>ACCTCTATCAGCGGTACCGAATCTGCTGACACCTACCTGTACGACCCGGCTGACCCGGTTCCGAGCCTGGGTGGT<br>ACCCTGCTGTTCCACAACGGTGACAACGGTCCGGCTGACCAGCGTCCGATCCACGACCGTGACGACGTTCTGTGC<br>TACTCTACCGAAGTTCTGACCGACCCGGTTGAAGTTACCGGTACCGTTTCTGCTCGTCTGTTCGTTTCTTCTTCTGC<br>TGTTGACACCGACTTCACCGCTAAACTGGTTGACGTTTTCCCGGACGGTCGTGCTATCGCTCTGTGCGACGGTATC<br>GTTCGTATGCGTTACCGTGAAACCCTGGTTAACCCGACCCTGATCGAAGCTGGTGAAATCTACGAAGTTGCTATCG<br>ACATGCTGGCTACCTCTAACGTTTTCCTGCCGGGTCACCGTATCATGGTTCAGGTTTCTTCTTCTAACTTCCCGAAA<br>TACGACCGTAACTCTAACACCGGTGGTGTTATCGCTCGTGAACAGCTGGAAGAAATGTGCACCGCTGTTAACCGTA<br>TCCACCGTGGTCCGGAACACCCGAGCCACATCGTTCTGCCGATCATCAAACGTTAA
**vdh**<br><br>*UniProtKB - D0RZT4*<br><br>Codon optimized and chemically synthesized	Aryl-aldehyde oxidase (**EC**: 1.2.3.9), *Acinetobacter johnsonii* SH046<br>Benzaldehyde to benzoate<br><br>ATGCACAACGTTCAGCTGAAACAGGACAACACCGTTGACACCTCTTCTTTCGAATCTGCTCCGAACGTTCACACCGT<br>TCAGCTGCTGATCCACGGTCAGTCTGTTGACGCTTCTAACCAGATGACCTTCAAACGTATCTCTCCGATCGACGGT<br>CAGGTTGCTTCTATCGCTGCTGCTGCTACCCTGGCTGACGTTGACCTGGCTATCGAATCTGCTGCTAAAGCTTTCC<br>CGATCTGGTCTAAACTGTCTCCGACCGAACGTCGTCTGCGTCTGCTGAAAGCTGCTGACCTGATGGACGCTCGTAC<br>CGACCAGTTCATCCAGATCGGTATGCGTGAAACCGGTTCTACCGCTACCTGGTACGGCTTCAACGTTCACCTCGCT<br>GCTAACATGCTGCGTGAAGCTGCTGCTATGACCACCCAGATGGACGGTTCTCTGATCCCGTCTGACGTTCCGGGTA<br>ACATGGCTATGGGTATCCGTGTTCCGTGCGGTGTTGTTGTTGGTATCGCTCCGTGGAACGCTCCGGTTATCCTGCC<br>GACCCGTGCACTGGCTATGCCGCTGGCTTGCGGTAACACCGTTGTTCTGAAAGCTTCTGAAGCTTGCCCGGCTAC<br>CCAGCGTCTGATCGGTCAGGTTCTGCACGAAGCTGGTCTGGGTGACGGTGTTGTTAACGTTATCACCCACGCTGCT<br>GAAGACGCTTCTCAGATCGTTGAACGTCTGATCTCTCACCCGGCTGTTAAACGTATCAACTTCACCGGTTCTACCAA<br>CGTTGGTAAAATCATCGCTGAAACCGCTGCTAAATACCTGAAACCGGTTCTGCTGGAACTGGGTGGTAAAGCTCCG<br>GTTGTTGTTCTGAACGAAGCTGACGTTGACGAAGCTGTTAACGCTGTTGTTTTCGGTGCTTTCTTCAACCAGGGTCA<br>GATCTGCATGTCTACCGAACGTGTTCTGGTTCAGGACCGTATCGCTGACCAGTTCATCGAAAAACTGATCGAAAAAA<br>CCCGTACCATCCACGCTGGTAACCCGACCTTCAAAGGTCACGTTCTGGGTGTTCTGGAATCTCAGCGTGCTGCTAA<br>CCGTATCCAGCACCTGCTGGAAGACGCTCAGTCTCAGGGTGCTGACCTGCCGCTGGGTATCCACATCCAGAACAC<br>CACCATGCAGCCGACCCTGGTTCTGAACATCCAGCCGGAAATGCTGCTGTACCGTGAAGAATCTTTCGGTCCGGTT<br>TGCACCGTTCAGCGTTTCAACTCTGTTGAAGAAGGTATCGCCCTGGCTAACGACTCTGAATTCGGTCTGTCTGCTG<br>CTGTTTTCTCTCAGGACATCGCTCAGGCCCTGGACGTTGCTAAACAGATCGACTCCGGTATCTGCCACATCAACGG<br>TGCTACCGTTCACGACGAAGCTCAGATGCCGTTCGGTGGTACCAAAGCTTCTGGTTACGGTCGTTTCGGTTCTAAA<br>GCTTCTATCGCTGAATTCACCGAACTGCGTTGGATCACCATCCAGACCCAGTCTCGTCACTACCCGATCTAA
**bphC**<br><br>*UniProtKB - P17297*<br><br>Codon optimized and chemically synthesized	Biphenyl-2,3-diol 1,2-dioxygenase (**EC**: 1.13.11.39), *Pseudomonas sp.*<br>Biphenyl-2,3-diol to 2-hydroxy-6-oxo-6-phenylhexa-2,4-dienoate<br><br>ATGAGCATTGAACGCTTAGGTTACCTGGGTTTCGCAGTGAAAGATGTGCCAGCCTGGGACCACTTTCTGACGAAAT<br>CCGTGGGCTTAATGGCGGCCGGTAGCGCCGGAGATGCAGCCCTTTACCGTGCGGACCAACGTGCTTGGCGCATC<br>GCAGTACAACCTGGTGAGCTTGACGATTTAGCCTATGCAGGCTTAGAGGTGGACGACGCAGCTGCGCTTGAACGT<br>ATGGCGGACAAATTACGTCAAGCTGGTGTTGCGTTCACCCGTGGGGACGAGGCCCTGATGCAACAGCGCAAAGTG<br>ATGGGGCTTCTTTGCTTGCAGGATCCATTTGGATTACCTTTGGAAATCTATTATGGACCTGCTGAAATTTTCCACGAA<br>CCATTCTTGCCGTCTGCTCCTGTTTCCGGGTTCGTGACCGGGGACCAGGGTATTGGCCATTTTGTCCGTTGTGTTC<br>CCGATACAGCGAAGGCTATGGCTTTTTACACCGAGGTCCTTGGGTTCGTGCTTTCAGACATTATTGACATTCAAATG<br>GGGCCCGAGACTTCCGTTCCCGCTCACTTCTTACATTGCAACGGACGCCATCACACTATCGCTTTGGCCGCCTTTC<br>CCATTCCGAAACGTATCCACCACTTCATGTTACAGGCAAACACAATCGACGACGTGGGTTACGCATTTGATCGTCTG<br>GATGCAGCAGGGCGCATTACCTCGCTGCTGGGGCGTCACACCAATGATCAGACCCTGAGCTTTTACGCTGATACC

	CCAAGCCCCATGATTGAGGTCGAATTCGGTTGGGGCCCGCGTACAGTGGATTCCTCTTGGACCGTAGCGCGTCAC<br>TCGCGCACCGCTATGTGGGGGCATAAGTCTGTTCGCGGACAACGCTAA
**bphD**<br><br>*UniProtKB - Q52036*<br><br>Codon optimized and chemically synthesized	2-Hydroxy-6-oxo-6-phenylhexa-2,4-dienoate hydrolase (**EC**: 3.7.1.8), *Pseudomonas putida*<br>2-hydroxy-6-oxo-6-phenylhexa-2,4-dienoate to benzoate<br><br>ATGACAGCATTGACTGAAAGCTCTACTAGCAAATTCCTTAACATCAAAGAGAAAGGCTTGTCCGACTTTAAGATTCAT<br>TATAATGAAGCGGGCAACGGTGAAACTGTCATCATGCTGCATGGCGGTGGACCGGGAGCCGGAGGATGGTCGAA<br>CTATTATCGTAATATCGGACCGTTCGTTGAAGCCGGTTACCGTGTCATTTTGAAGGATTCACCCGGCTTTAACAAAT<br>CCGATGCTGTCGTCATGGATGAACAACGTGGGCTTGTAAATGCTCGTGCGGTCAAGGGATTGATGGATGCTCTTGG<br>CATTGATCGTGCGCATCTGGTGGGAAATTCAATGGGAGGTGCAACCGCGCTTAACTTCGCCATCGAGTATCCAGAC<br>CGTATTGGAAAACTTATCCTTATGGGTCCGGGAGGTTTGGGACCCTCCATGTTTGCCCCAATGCCCTTAGAGGGAA<br>TTAAATTATTATTTAAGTTATATGCAGAGCCGTCGTATGAAAATCTGAAACAGATGATCCAAGTGTTCCTTTATGATCA<br>ATCTCTGATTACTGAGGAACTTTTACAAGGACGCTGGGAAGCCATTCAGCGTCAACCAGAACATCTTAAAAACTTCC<br>TGATTTCTGCGCAGAAGGCGCCCCTGAGTACGTGGGATGTTACCGCCCGTTTGGGAGAGATTAAGGCGAAGACCT<br>TCATTACATGGGGTCGTGACGACCGCTTCGTGCCGTTAGACCATGGTCTGAAACTTTTGTGGAATATTGATGACGC<br>ACGCTTGCACGTTTTTTCCAAGTGCGGACATTGGGCACAATGGGAGCATGCTGACGAGTTTAACCGCTTAGCCATT<br>GACTTTCTGCGCCAGGCTTAA
UniProtKB - B4XEY3<br><br>Codon optimized and chemically synthesized	Amidase (**EC**: 3.5.1.4), *Rhodococcus erythropolis*<br>Benzamide to benzoate<br><br>ATGGCGACAATCCGTCCCGATGACAACGCAATTGACACGGCGGCCCGCCATTATGGCATCACCCTTGACCAAAGC<br>GCGCGTCTTGAGTGGCCCGCACTTATTGACGGAGCCTTAGGGAGCTACGACGTTGTTGACCAGCTGTACGCTGAT<br>GAAGCCACGCCGCCAACAACGTCGCGTGAACATACTGTCCCTACTGCTAGCGAAAATCCCCTTTCCGCCTGGTAC<br>GTTACGACCTCTATCCCCCCCACAAGTGACGGAGTGTTGACTGGACGCCGCGTCGCCATCAAAGATAACGTCACA<br>GTAGCTGGCGTGCCAATGATGAACGGCTCGCGTACCGTTGAGGGATTTACTCCGTCACGCGACGCCACTGTAGTC<br>ACTCGCCTGCTGGCTGCTGGTGCAACAGTAGCTGGAAAGGCTGTCTGTGAGGACTTATGCTTTTCTGGCTCTAGTT<br>TTACCCCAGCCTCGGGACCTGTTCGCAATCCCTGGGATCCGCAGCGCGAGGCAGGAGGAAGTTCCGGCGGAAGT<br>GCAGCATTAGTAGCAAATGGCGATGTCGACTTCGCAATTGGAGGTGACCAGGGTGGCTCCATCCGTATCCCGGCT<br>GCCTTTTGCGGCGTAGTCGGCCACAAGCCTACATTTGGACTTGTACCATATACGGGAGCCTTCCCAATCGAACGCA<br>CGATTGACCACCTTGGACCGATTACACGCACTGTCCATGACGCTGCACTTATGCTGTCAGTTATCGCAGGCCGCGA<br>TGGAAACGACCCTCGTCAAGCGGATAGTGTGGAAGCGGGCGACTACCTTAGTACTTTAGATAGCGACGTCGACGG<br>GTTACGTATCGGAATCGTACGTGAGGGTTTTGGCCACGCAGTCAGCCAACCGGAGGTAGACGACGCGGTTCGTGC<br>AGCGGCTCACAGCTTAGCAGAAATCGGATGCACAGTGGAAGAAGTGAACATTCCATGGCACCTGCATGCGTTTCAT<br>ATCTGGAATGTGATTGCCACCGATGGCGGTGCTTACCAAATGTTAGACGGGAACGGTTATGGAATGAATGCAGAAG<br>GTTTATACGACCCTGAACTTATGGCTCACTTCGCATCTCGTCGTCTTCAACATGCAGATGCCTTGTCTGAAACCGTT<br>AAGCTTGTAGCTCTGACCGGCCACCACGGGATTACGACATTAGGGGGCGCTTCGTACGGGAAAGCCCGCAACTTG<br>GTTCCGTTAGCGCGTGCAGCTTACGACACCGCGCTTCGTCAGTTCGACGTGCTTGTAATGCCAACTTTACCTTATG<br>TCGCCTCAGAATTACCAGCCAATGATGTCGACCGTGCAACTTTTATTACTAAGGCGCTTGGTATGATCGCTAACACA<br>GCACCTTTCGATGTAACAGGGCACCCGAGCTTATCAGTTCCAGCTGGCCTTGTAAATGGGTTACCTGTCGGTATGA<br>TGATTACTGGAAAGACTTTTGATGATGCGACAGTGCTTCGTGTAGGGCGTGCCTTTGAGAAATTACGTGGGGCCTT<br>TCCGACCCCTGCAGATCACATTTCGGATAGTGCCCCGCAATTAAGCCCTGCGTAA
**J23101-B0032**<br><br>From iGEM registry[4]	Constitutive promoter-RBS<br><br>AGGATACTAGAGGATGACCCCATCTGTTTACAGCTAGCTCAGTCCTAGGTATTATGCTAGCTAGTAGAGTCACACAG<br>GAAAGTAGTAGATG

Supplementary Table 6. List of plasmids used in this study deposited to Addgene[5] available at:

Voyvodic et al.[6] https://www.addgene.org/browse/article/28196338/
This study https://www.addgene.org/browse/article/28203589/

Plasmids name	Description/Experimental Purpose	Addgene ID
pBEAST-BenR	Strong constitutive expression of transcription factor, BenR, for cell-free expression.	114597 (Voyvodic et al.[6])
pBEAST-pBen-sfGFP	Output expression of sfGFP under the activation of BenR transcription factor for cell-free expression	114598 (Voyvodic et al.[6])
pBEAST-J23101-CocE	Strong constitutive expression of metabolic enzyme, CocE, for cell-free expression	114600 (Voyvodic et al.[6])
pBEAST_J23101-bphD	The cell-free adapted backbone, pBEAST, expressing gene encoding bphD (the enzyme converting 2-hydroxy-6-oxo-6-phenylhexa-2,4-dienoate to benzoate) under control of the constitutive promoter J23101 and RBS B0032	128138 (This study)
pBEAST_J23101-bphC	The cell-free adapted backbone, pBEAST, expressing gene encoding bphC (the enzyme converting biphenyl-2,3-diol to 2-hydroxy-6-oxo-6-phenylhexa-2,4-) under control of the constitutive promoter J23101 and RBS B0032	128137 (This study)

pBEAST_J23101-amidase	The cell-free adapted backbone, pBEAST, expressing the amidase enzyme gene (benzamid to benzoate) under control of the constitutive promoter J23101 and RBS B0032	128135<br>(This study)
pBEAST_J23101-vdh	The cell-free adapted backbone, pBEAST, expressing gene encoding vdh (the enzyme converting benzaldehyde to benzoate) under control of the constitutive promoter J23101 and RBS B0032	128134<br>(This study)
pBEAST_J23101-HipO	The cell-free adapted backbone, pBEAST, expressing gene encoding HipO (the enzyme converting hippurate to benzoate) under control of the constitutive promoter J23101 and RBS B0032	128133<br>(This study)
pSB4C5_J23101-(B0032-HipO_B0034vdh)	Expressing genes encoding HipO and vdh in one operon under control of the constitutive promoter J23101, and RBS B0032 for HipO and RBS B0034 for vdh	128131<br>(This study)
pSB4C5_J23101-vdh	Expressing vdh gene (for the enzyme transforming benzaldehyde to benzoate) under control of the constitutive promoter J23101 and RBS B0032	128130<br>(This study)
pSB4C5_J23101-CocE	Expressing gene encoding CocE enzyme (cocaine to benzoate) gene under control of the constitutive promoter J23101 and RBS B0032	128129<br>(This study)

pSB4C5_J23101-HipO	Expressing gene encoding HipO enzyme (hippurate to benzoate) gene under control of the constitutive promoter J23101 and RBS B0032	128128 (This study)
pSB4C5_pBen-BenR	Expressing gene encoding BenR transcription factor gene under control of benzoate responsive promoter (pBen) in a feedback loop.	128127 (This study)
pSB1K3_pBen-sfGFP_J23101-mRFP	Expressing gene encoding sfGFP under control of benzoate responsive promoter (pBen) and expressing gene encoding mRFP under constitutive promoter J23101 and RBS B0032	128126 (This study)
pSB1K3_pBen-sfGFP_J23101-BenR	Expressing gene encoding sfGFP under control of benzoate responsive promoter (pBen) and expressing gene encoding BenR transcription factor gene under constitutive promoter J23101 and RBS B0032	128125 (This study)

Supplementary Table 7. The mean and standard deviation of the normalized data of whole-cell and cell-free data plotted in all figures and supplementary figures, and model simulated/predicted results associated with each experiment, also submitted as Source Data excel file.

**Open-loop actuator (Fig. 1c)**			
**Benzoate concentrations**	**Mean**	**sd**	**Model**
**0**	**137.1253**	**52.75396**	**129.5562**
**1**	**152.4295**	**26.17023**	**134.3022**
**10**	**196.3033**	**15.81854**	**228.7778**
**20**	**370.6038**	**52.07807**	**366.597**
**100**	**1340.749**	**104.5505**	**1345.377**
**200**	**1974.003**	**76.27541**	**1940.671**
**500**	**2401.962**	**116.8234**	**2471.769**
**1000**	**2702.137**	**58.75755**	**2658.252**

**Feedback-loop actuator (Supp. Fig. S1b)**		
**benzoate concentration**	**Mean**	**sd**
**0**	**176.7221**	**14.40118**
**1**	**175.5545**	**8.976066**
**10**	**186.5161**	**5.700804**
**20**	**175.8244**	**11.7473**
**100**	**176.3523**	**6.871175**
**200**	**186.8994**	**22.29161**

500	229.1743	24.9362
1000	256.361	26.27477

Hippurate transducer (Fig. 1d)					
Hippurate concentrations	NC	sd	Mean	sd	Model
0	10.42038	10.42038	33.83452	5.982626	138.3214
1	9.230474	9.230474	36.76217	5.931294	142.0044
10	9.794407	9.794407	178.6825	20.18181	216.074
20	10.39639	10.39639	392.1922	59.44664	326.7622
100	11.44233	11.44233	1170.904	136.9077	1215.649
200	10.28643	10.28643	1595.289	337.6722	1863.389
500	13.43539	13.43539	2364.503	432.4425	2529.084
1000	14.14902	14.14902	2691.25	555.3749	2786.039

Cocaine transducer (Fig. 1e)					
Cocaine concentrations	NC	sd	Mean	sd	Model
0	2.578523	0.964539	0.699758	1.519025	106.7959
1	3.795796	1.281066	1.083956	0.890681	107.2539
10	5.247815	0.932223	22.44099	4.644204	116.6802
20	5.259497	0.517627	77.24693	13.89922	131.5785
100	5.967215	1.530721	428.5773	131.4049	302.8264

200	5.396151	1.450211	711.0437	98.96636	542.4661
500	9.127592	1.647522	1208.372	175.431	1110.959
1000	22.80564	4.480886	1329.617	76.54072	1601.437

Benzaldehyde transducer (Fig. 1f)					
Benzaldehyde concentrations	NC	sd	Mean	sd	Model
0	2.873426	0.87706	68.18518	24.74003	106.7959
1	3.840284	1.429621	100.9618	37.40521	111.2383
10	4.301073	0.731954	303.4843	122.2295	199.2261
20	4.107255	0.917198	453.409	61.38622	326.0815
100	22.17864	1.96911	1167.718	277.1315	1178.782
200	47.23322	7.509535	1436.268	412.567	1659.353
500	157.5873	22.40705	1970.066	69.83603	2066.138
1000	433.0743	76.723	2103.431	74.13477	2204.35

Hippurate-benzaldehyde concentration adder (Fig. 2b and 2c, in vivo and model data)					
	Hippurate concentrations	Benzaldehyde concentrations	model	Mean	sd
1	0	0	48.39032	47.51496	34.85855
2	0	1	50.40322	27.97123	28.93989
3	0	10	90.27141	84.88917	59.20592
4	0	20	147.7509	158.3545	92.4153
5	0	100	534.1186	475.0621	185.3318

6	0	500	936.1886	903.2327	213.803
7	0	1000	998.8138	919.1106	213.8193
8	1	0	49.6788	30.05882	19.74518
9	1	1	52.52181	28.19774	29.37242
10	1	10	94.13735	93.33507	56.48188
11	1	20	152.0326	159.4401	84.51181
12	1	100	536.491	381.3766	79.87722
13	1	500	936.3736	732.908	122.5856
14	1	1000	998.8558	1166.612	236.5423
15	10	0	75.59127	86.21446	50.38076
16	10	1	80.61638	81.19441	49.33897
17	10	10	130.8714	139.937	76.1301
18	10	20	190.7461	188.635	96.26744
19	10	100	557.122	470.0044	173.4254
20	10	500	938.0144	804.201	224.0854
21	10	1000	999.231	1134.184	295.3284
22	20	0	114.3144	175.0692	115.3943
23	20	1	120.0905	124.1758	84.0242
24	20	10	173.6536	231.4451	163.5917
25	20	20	233.2806	273.8019	134.089
26	20	100	578.5992	463.7883	134.5797

27	20	500	939.7882	704.2105	44.10476
28	20	1000	999.6417	1063.241	377.3755
29	100	0	425.2822	597.2984	288.7776
30	100	1	429.6361	470.3275	285.0136
31	100	10	467.0102	490.0771	278.2035
32	100	20	504.9083	587.1758	308.1422
33	100	100	707.7917	557.0478	123.6608
34	100	500	952.3317	930.1619	287.9087
35	100	1000	1002.709	1092.143	349.8937
36	500	0	884.7742	862.9617	369.9712
37	500	1	885.2747	794.7597	190.4564
38	500	10	889.6618	877.49	145.7884
39	500	20	894.3008	938.5556	119.4559
40	500	100	924.2587	1036.408	163.6042
41	500	500	988.921	1181.836	208.2064
42	500	1000	1013.8	1270.341	369.709
43	1000	0	974.6671	886.9526	220.3447
44	1000	1	974.7888	891.2346	131.7974
45	1000	10	975.8681	899.482	134.6977
46	1000	20	977.034	1087.89	166.0846
47	1000	100	985.2558	1111.723	233.7399

48	1000	500	1009.024	1158.32	274.9251
49	1000	1000	1021.962	1478.605	287.9171

Benzoate actuator (Fig. 3b)			
Benzoate Concentrations	Data mean	Data sd	Model
0	0.033011438	0.007420496	0.032948286
1	0.054448326	0.000653338	0.075157603
5	0.485035272	0.128358282	1.12905611
10	3.21651485	0.14101149	2.723713493
50	4.241992557	0.174111638	4.496461865
100	4.673264388	0.159454201	4.605450067
500	5.017578705	0.074886371	4.951792779
1000	5.259845216	0.156300164	5.361737472

Hippurate transducer (Fig. 3c)				
Hippurate concentration	NC	sd	Data Means	Data Sd
0	0.01859	0.013555	0.018196	0.003558
10	0.028282	0.007689	1.203237	0.168961

100	0.037257	0.004361	3.943558	0.183397
1000	0.061559	0.009436	4.414297	0.484822

Cocaine transducer (Fig. 3d)

Cocaine concentration	NC	sd	Data Means	Data Sd
0	0.02859	0.012555	0.017033	0.003252
10	0.025282	0.007689	0.592297	0.475485
100	0.036257	0.004361	2.632578	0.463412
1000	0.055559	0.009436	3.42496	0.582069

Benzaldehyde transducer (Fig. 3e)

Benzaldehyde concentration	NC	sd	Data Means	Data Sd
0	0.051592	0.007427	0.07209	0.04227
10	0.204802	0.034533	0.747988	0.26426
100	3.199616	0.08219	3.661972	0.166329
1000	4.784759	0.160701	4.322671	0.149633

Benzamide transducer (Fig. 3f)

Benzamide concentration	NC	sd	Data Means	Data Sd
0	0.051592	0.007427	0.07209	0.04227
10	0.043164	0.009378	2.761356	0.099712
100	0.118696	0.023099	4.299468	0.11708
1000	0.585144	0.079395	3.977133	0.067883

Biphenyl-2,3-diol transducer (Fig. 3g)								
Biphenyl-2,3-diol concentration	NC	sd	Data Means	Data Sd	only enzyme 1	sd	only enzyme 2	sd
0	0.032658	0.004461	0.032591	0.006763	0.032886	0.004461	0.033712	0.014259
10	0.039945	0.01463	0.10021	0.03997	0.041163	0.02168	0.040885	0.018025
100	0.036436	0.015096	3.45308	0.32505	0.038145	0.023125	0.04936	0.025325
1000	0.026511	0.002102	2.39105	0.332053	0.031618	0.003012	0.030489	0.003325

Hippurate weighted transducer (Fig. 4b)				
Hippurate concentration	HipO [nM]	Data Means	Data Sd	Model
0	0.1	0.00818244	0.00139968	0.03075614
10	0.1	0.65776276	0.04402834	0.05689169
100	0.1	2.57263017	0.10171441	2.24348368
1000	0.1	3.40759119	0.08937716	3.80489409
0	0.3	0.00811206	0.00282825	0.03075614
10	0.3	1.3414102	0.10062636	0.66883489
100	0.3	3.28084253	0.05991059	3.95271157
1000	0.3	3.80353341	0.07346332	4.13883002
0	1	0.00336096	0.00195845	0.03075614
10	1	2.54224076	0.16906574	2.06598955
100	1	3.68595259	0.14276648	4.08654164
1000	1	4.33864752	0.1752886	4.30217219

0	3	0.00848159	0.00459283	0.03075614
10	3	2.49515212	0.02119017	2.3672994
100	3	4.11198508	0.1491968	4.10493864
1000	3	4.55381935	0.04947948	4.34647867
0	10	0.00721463	0.00211972	0.03075614
10	10	2.49062978	0.1654227	2.41173608
100	10	4.00351933	0.03257552	4.10775299
1000	10	4.5330905	0.05971498	4.35371225

Cocaine weighted transducer (Fig. 4c)				
Cocaine concentration	CocE [nM]	Data Means	Data Sd	Model
0	0.1	0.00783051	0.00331458	0.03075614
10	0.1	0.56926921	0.05623263	0.05585811
100	0.1	1.57792676	0.2993573	1.52850911
1000	0.1	1.67344138	0.29497577	2.21612192
0	0.3	0.00703866	0.00199929	0.03075614
10	0.3	1.22031005	0.17895399	0.801576
100	0.3	2.69981875	0.12090086	3.88620162
1000	0.3	2.85549631	0.12248131	3.97994033
0	1	0.00823523	0.00562331	0.03075614
10	1	1.44253814	0.07074442	3.21817476
100	1	3.42455436	0.03567821	4.12606657

1000	1	3.34734027	0.03489832	4.1766156
0	3	0.00834081	0.00117569	0.03075614
10	3	1.56789667	0.15795989	3.6579708
100	3	3.82947087	0.39735051	4.19217237
1000	3	3.76300832	0.03985681	4.26325038
0	10	0.00761935	0.00335635	0.03075614
10	10	1.65839624	0.0614677	3.71767934
100	10	3.63471115	0.18105836	4.20846933
1000	10	3.82883739	0.23721058	4.28533365

Benzamide weighted transducer (Fig. 4d)				
Benzamide concentration	Enzyme [nm]	Data Means	Data Sd	Model
0	0.1	0.04220047	0.00435683	0.03075614
10	0.1	1.41967756	0.18146775	0.58093365
100	0.1	2.22916367	0.15121954	2.7412603
1000	0.1	2.18053356	0.06430761	3.29966523
0	0.3	0.04071302	0.01579984	0.03075614
10	0.3	2.0535243	0.15796188	1.6023616
100	0.3	3.0744446	0.06747095	3.62095339
1000	0.3	3.02691809	0.01510055	3.8385399
0	1	0.03785807	0.01060016	0.03075614
10	1	2.47790413	0.19194935	2.89476998

100	1	3.65903747	0.24619976	3.94075294
1000	1	3.00772516	0.22694437	4.01705851
0	3	0.03740224	0.00908132	0.03075614
10	3	2.51796939	0.22721728	3.52314283
100	3	3.6559666	0.55775483	4.03521326
1000	3	3.28496713	0.20119771	4.08316964
0	10	0.03363562	0.00845691	0.03075614
10	10	1.92860227	0.24099681	3.7854101
100	10	3.61405403	1.07598812	4.08345856
1000	10	2.92034931	0.52051559	4.12981517

Biphenyl-2,3,diol weighted transducer (Fig. 4e)				
Biphenyl-2,3-diol concentration	Enzyme [nM]	Data Means	Data Sd	Model
0	0.1	0.04791037	0.01207362	0.03075614
10	0.1	0.03557891	0.01257012	0.03075718
100	0.1	0.03821794	0.02057939	0.03257536
1000	0.1	0.05374022	0.02268666	0.03326166
0	0.3	0.0418406	0.00544404	0.03075614
10	0.3	0.0342354	0.00946892	0.03076068
100	0.3	0.05282856	0.01902893	0.06004588
1000	0.3	0.04263231	0.00621908	0.07144537
0	1	0.03814596	0.00687827	0.03075614

10	1	0.04745454	0.01676262	0.03078609
100	1	0.58461686	0.3580844	0.56334937
1000	1	1.19715945	0.08701882	0.73756654
0	3	0.04601507	0.02141733	0.03075614
10	3	0.1038098	0.03477485	0.03097656
100	3	2.68621947	0.15598616	2.65903268
1000	3	1.823377	0.72915661	2.94486015
0	10	0.05254067	0.00957248	0.03075614
10	10	0.11484574	0.04520265	0.03211607
100	10	2.93037762	0.30506833	3.78146507
1000	10	2.34696032	0.46910023	3.85731348

Fixed-input adder (Fig. 5b)				
HipO [nM]	CocE [nM]	Data Means	Data Sd	Model
0	0	0.01557544	0.00744527	0.03075614
0	0.1	0.7363064	0.06655886	1.52850911
0	0.3	2.71275387	0.20333374	3.88620162
0	1	3.92735407	0.23505573	4.12606657
0	3	4.3966056	0.23787075	4.19217237
0	10	4.41544762	0.13869244	4.20846933
0.1	0	2.0035743	0.35953586	2.24348368
0.1	0.1	2.5951096	0.24460087	3.20307532

**0.1**	**0.3**	**3.30445486**	**0.32274965**	**3.95395775**
**0.1**	**1**	**4.1505468**	**0.5274273**	**4.13491436**
**0.1**	**3**	**4.37186953**	**0.3436348**	**4.20007061**
**0.1**	**10**	**4.44330824**	**0.135122**	**4.21628257**
**0.3**	**0**	**3.40846471**	**0.53682725**	**3.95271157**
**0.3**	**0.1**	**3.80703499**	**0.30331647**	**3.98346329**
**0.3**	**0.3**	**3.8872556**	**0.34385447**	**4.05846747**
**0.3**	**1**	**4.2076883**	**0.16863396**	**4.16847258**
**0.3**	**3**	**4.54940113**	**0.17667821**	**4.23130119**
**0.3**	**10**	**4.88706623**	**0.18654025**	**4.24728003**
**1**	**0**	**4.2720731**	**0.28952273**	**4.08654164**
**1**	**0.1**	**4.51903139**	**0.1540308**	**4.09426971**
**1**	**0.3**	**4.21438716**	**0.17078485**	**4.12633558**
**1**	**1**	**4.54199214**	**0.29444058**	**4.21784173**
**1**	**3**	**4.57543909**	**0.14201456**	**4.27915312**
**1**	**10**	**4.78379018**	**0.19123379**	**4.29495066**
**3**	**0**	**4.50312456**	**0.15692217**	**4.10493864**
**3**	**0.1**	**5.07669365**	**0.04954622**	**4.11182082**
**3**	**0.3**	**4.73401032**	**0.14025071**	**4.14164622**
**3**	**1**	**4.76745727**	**0.29080678**	**4.23142483**
**3**	**3**	**5.04050088**	**0.10178196**	**4.29250735**

3	10	5.02191924	0.16487221	4.30827422
10	0	4.86533636	0.03951931	4.10775299
10	0.1	4.92018179	0.04086461	4.11453635
10	0.3	4.91873787	0.02371656	4.1440891
10	1	4.80530701	0.0140498	4.23363984
10	3	4.80684562	0.17977087	4.29468972
10	10	4.99640203	0.09160087	4.31045211

**Fixed-enzyme adder (Fig. 5c)**				
**Cocaine concentration**	**Hippurate concentration**	**Data Means**	**Data Sd**	**Model**
0	0	0.02890776	0.01229848	0.03075614
1	0	2.50704813	0.29159956	0.07282929
10	0	3.72227686	0.27774708	3.6579708
20	0	4.23504694	0.29713403	4.00216523
100	0	4.25802414	0.43255079	4.19217237
500	0	4.37153926	0.38333854	4.25709675
1000	0	4.20253315	0.29124023	4.26325038
0	1	0.14307853	0.02221961	0.04382733
1	1	2.45614662	0.21623354	0.1363302
10	1	3.71735956	0.31739458	3.67413653
20	1	4.03132171	0.25105141	4.00387789
100	1	4.10883624	0.044408	4.19263535

500	1	4.26928923	0.38938268	4.25754555
1000	1	4.13735658	0.65555047	4.26369852
0	10	2.70081955	0.42154458	2.06598955
1	10	3.28219341	0.37028665	2.28823759
10	10	4.32680674	0.15476818	3.83457333
20	10	4.06705409	0.23903666	4.0256109
100	10	4.48165698	0.52572889	4.19943036
500	10	4.35103561	0.08582767	4.26414588
1000	10	4.4131128	0.45672211	4.27028957
0	20	3.55637014	0.08878817	3.43685339
1	20	3.84520936	0.24686224	3.48806007
10	20	3.62735807	0.65475503	3.93533938
20	20	4.0115333	0.33261698	4.04756933
100	20	4.22232156	0.26069877	4.20841057
500	20	4.13085978	0.49486215	4.27290328
1000	20	4.02405007	0.79020601	4.27903609
0	100	4.30838921	0.33962656	4.08654164
1	100	4.38766205	0.18129735	4.08769648
10	100	4.38730443	0.1179161	4.11345613
20	100	4.27849799	0.50736451	4.14590554
100	100	4.55336016	0.18226843	4.27915312

500	100	4.13378036	0.32106185	4.34269266
1000	100	4.26711369	0.19703681	4.3487746
0	500	4.37261213	0.30074501	4.25394378
1	500	4.4606765	0.56314037	4.25473605
10	500	4.31503502	0.35309432	4.2740985
20	500	4.82026524	0.34336437	4.3019407
100	500	4.47960066	0.23051345	4.43006894
500	500	4.06151095	0.43288144	4.49309776
1000	500	4.24771271	0.58311348	4.49914761
0	1000	4.46908061	0.41765843	4.30217219
1	1000	4.30874683	0.4911537	4.30295735
10	1000	4.45465653	0.65667822	4.32217003
20	1000	4.48156758	0.49738212	4.34985842
100	1000	4.33887647	0.23101415	4.47768932
500	1000	4.46812696	0.33022075	4.54066601
1000	1000	4.12260468	0.42281116	4.54671219

Full-OR classifier			
**Inputs**	**Data Means**	**Data Sd**	**Model**
No input	0.035304	0.012647	0.0307561435577849
H	3.88545	0.224492	4.12606656849739
C	3.249831	0.164483	4.08654164331305

B	3.739878	0.05422	3.94075294149016
F	3.136258	0.14312	3.78146507270607
HC	4.188237	0.139133	4.2178417281617
HB	3.979569	0.173847	4.16667903537404
HF	3.915947	0.217096	4.1535358879735
CB	3.542327	0.016789	4.1330352706629
BF	3.798092	0.066124	4.04111973849995
CF	3.513107	0.064933	4.11868545369628
HCB	4.075715	0.04238	4.25481654344573
HCF	3.98986	0.028954	4.24253751407664
HBF	3.729362	0.208663	4.19226127860961
CBF	4.034102	0.204885	4.1600418007154
HCBF	3.897919	0.056789	4.27923889568576

(C AND H) OR B OR F classifier			
Inputs	Data Means	Data Sd	Model
No input	0.022609	0.00315	0.0307561435577849
H	1.182528	0.097834	1.52850911478862
C	0.610832	0.04898	0.0874951181769892
B	3.827637	0.100457	3.94075294149016
F	3.411953	0.09547	3.78146507270607
HC	1.769599	0.135243	1.84515671973946

**HB**	**3.703373**	**0.050647**	**3.9749354664424**
**HF**	**3.27184**	**0.092088**	**3.87366620345265**
**CB**	**3.676482**	**0.174047**	**3.94739701632846**
**BF**	**3.837803**	**0.04878**	**4.04111973849995**
**CF**	**2.982899**	**0.048738**	**3.80078972620523**
**HCB**	**3.585393**	**0.184831**	**3.97965207156987**
**HCF**	**3.558552**	**0.387636**	**3.88501043634454**
**HBF**	**3.842735**	**0.124697**	**4.0530838122063**
**CBF**	**3.860462**	**0.107688**	**4.04326124317119**
**HCBF**	**3.840582**	**0.147427**	**4.05494186068752**

Supplementary References:

1. Daniel, R., Rubens, J. R., Sarpeshkar, R. & Lu, T. K. Synthetic analog computation in living cells. *Nature* **497**, 619–623 (2013).
2. Libis, V., Delépine, B. & Faulon, J.-L. Expanding Biosensing Abilities through Computer-Aided Design of Metabolic Pathways. *ACS Synth. Biol.* **5**, 1076–1085 (2016).
3. Cowles, C. E., Nichols, N. N. & Harwood, C. S. BenR, a XylS homologue, regulates three different pathways of aromatic acid degradation in Pseudomonas putida. *J. Bacteriol.* **182**, 6339–6346 (2000).
4. parts.igem.org. Available at: http://parts.igem.org/Main_Page.
5. Addgene. Available at: http://www.addgene.org/.
6. Voyvodic, P.L., Pandi, A., Koch, M., Conejero, I., Valjent, E., Courtet, P., Renard, E., Faulon & J.L. and Bonnet, J. Plug-and-play metabolic transducers expand the chemical detection space of cell-free biosensors. *Nat. Commun.* **10**, 1697 (2019).
